# Radiotherapy of prostate cancer: impact of treatment characteristics on the incidence of second tumors

**DOI:** 10.1186/s12885-020-6581-5

**Published:** 2020-02-03

**Authors:** Milly BUWENGE, Erica SCIROCCO, Francesco DEODATO, Gabriella MACCHIA, Maria NTRETA, Silvia BISELLO, Giambattista SIEPE, Savino CILLA, Anna Rita ALITTO, Vincenzo VALENTINI, Lidia STRIGARI, Alessio G. MORGANTI, Silvia CAMMELLI

**Affiliations:** 1 Radiation Oncology Center, Department of Experimental, Diagnostic and Specialty Medicine – DIMES, University of Bologna, S. Orsola-Malpighi Hospital, via Giuseppe Massarenti 9, 40138 Bologna, Italy; 2 Radiotherapy Unit, Gemelli Molise Hospital, Catholic University of Sacred Heart, Campobasso, Italy; 3 Medical Physic Unit, Gemelli Molise Hospital, Catholic University of Sacred Heart, Campobasso, Italy; 4 Fondazione Policlinico Universitario “A. Gemelli” IRCCS, UOC Radioterapia Oncologica, Dipartimento di Diagnostica per Immagini, Radioterapia Oncologica ed Ematologia, Rome, Italy; 5 Università Cattolica del Sacro Cuore, Istituto di Radiologia, Rome, Italy; 6 Medical Physics Unit, “S. Orsola-Malpighi” Hospital, Bologna, Italy

**Keywords:** Second malignancy, 3D-conformal radiotherapy, Intensity modulated radiotherapy, Volumetric modulated arc therapy, Prostate neoplasms

## Abstract

**Background:**

It has been hypothesized that radiotherapy (RT) techniques delivering radiations to larger volumes (IMRT, VMAT) are potentially associated with a higher risk of second primary tumors. The aim of this study was to analyse the impact of RT technique (3D-CRT vs IMRT/VMAT) on the incidence of second tumors in prostate cancer (PCa) patients.

**Methods:**

A retrospective study on 2526 previously irradiated PCa patients was performed. Patients were treated with 3D-CRT (21.3%), IMRT (68.1%), or VMAT (10.6%). Second tumors incidence was analysed in 3 categories: pelvic, pelvic and abdominal, and “any site”. The correlation with RT technique was analysed using log-rank test and Cox’s proportional hazard method.

**Results:**

With a median follow-up of 72 months (range: 9–185), 92 (3.6%) cases of second tumors were recorded with 48 months (range: 9–152) median interval from RT. Actuarial 10-year second tumor free survival (STFS) was 87.3%. Ten-year STFS in patients treated with 3D-CRT and IMRT/VMAT was 85.8 and 84.5%, respectively (*p: .627*). A significantly higher 10-year cumulative incidence of second tumors in the pelvis was registered in patients treated with IMRT/VMAT compared to 3D-CRT (10.7% vs 6.0%; *p: .033*). The lower incidence of second pelvic cancers in patients treated with 3D-CRT was confirmed at multivariable analysis (HR: 2.42, 95%CI: 1.07–5.47, *p: .034*).

**Conclusions:**

The incidence of second pelvic tumors after RT of PCa showed a significant correlation with treatment technique. Further analyses in larger series with prolonged follow-up are needed to confirm these results.

## Background

Prostate cancer (PCa) is the second most common cancer in men worldwide [1]. In the USA, data from the Surveillance, Epidemiology and End Results database led to a forecast of approximately 174,650 new diagnoses and 31,620 deaths from PCa in 2019 [2].

Radiotherapy (RT) has been used in the treatment of PCa for over 70 years. RT results have gradually improved over time thanks to the technological evolution and to the combination with adjuvant androgen deprivation therapy (ADT). However, some studies suggested that patients undergoing RT show a slightly higher incidence of second primary tumors particularly in the pelvis [3–5], although other authors attributed this increased risk to other factors such as age and lifestyle [6].

In the late 1990s, 3-dimensional conformal RT (3D-CRT) emerged as the optimal RT technique for this tumor due to improved dose distribution compared to conventional 2-dimensional RT. In fact, a significant reduction of acute and late toxicity was demonstrated [7, 8]. In the following decade, 3D-CRT was progressively replaced in this setting by modulated RT techniques such as intensity-modulated RT (IMRT) first, and volumetric modulated arc therapy (VMAT) subsequently. In fact, these techniques allow a higher dose conformity due to the steeper dose gradients around the target volume, reduced irradiation of organs at risk (OAR), and therefore the delivery of higher RT doses to the tumor [9–13]. A meta-analysis showed that IMRT, compared to 3D-CRT, can achieve lower G2–4 rectal toxicity rates and improve biochemical relapse-free survival [14].

However, it is well known that modulated RT techniques lead to low-level doses in larger body volumes compared to 3D-CRT. Theoretically, this characteristic could increase the risk of RT-induced carcinogenesis and then of second tumors. The theoretically increased risk of IMRT/VMAT induced second tumors in PCa patients has been largely discussed in literature. Several studies addressed this topic mainly in planning and dosimetric analyses [15–18]. However, comparisons between 3D-CRT and modulated RT techniques in terms of second tumors incidence based on real clinical data are still lacking.

Therefore, the aim of this retrospective analysis was to evaluate the impact of RT technique (3D-CRT vs IMRT/VMAT) on the incidence of second primary tumors in PCa patients. Moreover, also the impact of ADT and irradiated volumes in terms of delivery or not of prophylactic nodal irradiation (PNI), was investigated.

## Methods

### End points and study design

The primary end point of this study was the correlation of RT technique with second primary cancers incidence in PCa. The secondary objectives of the analysis were the correlation of ADT and PNI on the same outcome. The study design was a monocentric retrospective analysis on all PCa patients previously treated with external beam RT (EBRT) included in our institutional PCa database.

### Inclusion criteria

Inclusion criteria were as follows: 1) histologically confirmed prostatic adenocarcinoma; 2) curative aim of RT; 3) age > 18 years. Exclusion criteria were: 1) patients with distant metastases; 2) palliative aim of RT; 3) previous chemotherapy or RT on any site of the body; 4) some diseases potentially affecting tolerance to radiation therapy and potentially associated to a higher risk of cancer: ulcerative colitis, Crohn’s disease, familial adenomatous polyposis, and bladder papilloma; 5) patients with malignancies diagnosed prior to PCa diagnosis; 6) patients with malignancies diagnosed during PCa staging and planning.

### Radiotherapy

All patients underwent computed tomography (CT) simulation in supine position using a personalized immobilization system. In some patients, Positron Emission Tomography (18F-choline or 11C-choline or 68-Ga-PSMA) - CT simulation and/or CT-simulation image fusion with MRI scans were performed. The Clinical Target Volumes (CTV) were defined based on risk categories to include only the prostate (or prostatic bed) +/− seminal vesicles or also pelvic lymph nodes. An isotropic margin ranging between 5 and 10 mm was added to the CTV to define the Planning Target Volumes. The photon beam energy was 10–15 MV and 6 MV in patients treated with 3D-CRT and IMRT/VMAT, respectively. As previously described, daily set-up verification was performed using an Electronic Portal Imaging Device in most patients [19]. Only in a small minority of patients treated after 2016, set-up and organ motion evaluation was performed using a cone-beam CT. Dose specification and prescription were performed based on the International Commission of Radiation Unit reports 62 and 83 for 3D-CRT and IMRT/VMAT techniques, respectively [20, 21]. ADT was prescribed according to risk categories.

### Follow-up

Patients were monitored weekly during RT. For a more precise estimate of acute toxicity, patients were evaluated at one month after the end of RT. Subsequently the patients were followed biannually for the first 5 years and annually thereafter. At each visit total PSA, blood count and urine test were requested. During each visit, the patients underwent rectal exploration and an interview on any symptoms that appeared in previous months. In the case of at least 2 episodes of rectal bleeding, the patients were referred to proctoscopy and, in cases of persistent bleeding, to total colonoscopy. In the case of microscopic or macroscopic hematuria, in the absence of clear signs of inflammation, patients were asked to undergo cystoscopy. In the case of recurrent bleeding, a renal and ureteral ultrasound was prescribed. Overall, 193 patients were lost at follow-up.

### Statistical analysis

The IBM SPSS Version 22.0 software package was used for statistical computation (IBM Corp, Armonk, NY, USA). Survival estimates were calculated by the Kaplan-Meier product-limit method and compared with the log-rank test. Multivariate analysis was performed using a Cox regression model [22]. Also a Fine and Gray competing risk survival regression analysis was performed in R (version 3.5.2) using the library package “survival” and “cmprsk” to consider deaths from other causes as competing events. A *p* < 0.05 value was considered statistically significant. The impact of RT technique (3D-CRT vs IMRT/VMAT), ADT (yes or not), and PNI (yes or not) on the incidence of second primary tumors was estimated. Second tumors incidence was evaluated not only as “any second tumor” detected during the follow-up but also considering other 2 groups: i) second tumors in the pelvis and ii) second tumors in the abdomen or pelvis. In cases of doubtful interpretation of the information contained in the database for the purposes of this stratification, the diagnostic images of the second tumor were analysed.

### Ethical issues

The local institutional review board approved this analysis (311/2019/Oss/AOUBo, ICAROS-1 study). Only patients who had provided a written informed consent to the scientific use of their data were included.

## Results

### Patients characteristics

We included in the analysis 2526 PCa patients who met the inclusion criteria and received EBRT between 2002 and 2018. Median follow-up was 72 months (range: 9–185 months) and median age was 71 years (range: 43–93 years). The RT settings were definitive (54.2%), adjuvant (32.8%), or salvage treatment (13.0%). Patients were treated with 3D-CRT technique (21.3%), IMRT (68.1%), or VMAT (10.6%). Total 3D-CRT median delivered dose was 70 Gy (median dose/fraction: 2.5 Gy) and the total IMRT/VMAT median dose was 67.5 Gy (median dose/fraction: 2.6 Gy). PNI and ADT were prescribed to 1294 (51.2%) and 1689 (66.9%) patients, respectively. Patients treated with 3D-CRT and IMRT/VMAT received PNI in 39.4 and 54.4% of cases, respectively.

### Incidence of second tumors

Ninety-two (3.6%) cases of second tumors were recorded. Median interval between RT and second tumor was 48 months (range: 9–152 months) and median age was 70 years (range: 45–83 years) at diagnosis of the second cancer. Moreover, there were 31 (1.2%), 26 (1.0%), and 35 (1.4%) cases of second primary cancers detected in the pelvis, abdomen, and other sites, respectively. Considering the group of younger patients (≤ 66 years: first quartile), we recorded 25 s tumors out of 688 cases. This information on second tumors was collected from patient chart-records. Table 1 shows the number and percentages of detected second tumors. The 10-year actuarial cumulative incidence of second tumors was 14.4%.

**Table 1:** Number and crude percentages of detected second tumors

Incidence of second tumors	Technique			Total 2526 (%)
	3D-CRT 538 (%)	IMRT 1719 (%)	VMAT 269 (%)	
No	515 (95.7)	1660 (96.6)	259 (96.3)	2434 (96.4)
Pelvis				
Bladder	8 (1.5)	19 (1.1)	4 (1.5)	31 (1.2)
Rectum	0 (0.0)	4 (0.2)	1 (0.4)	5 (0.2)
Sigma	0 (0.0)	2 (0.1)	0 (0.0)	2 (0.1)
Abdomen				
Colon	0 (0.0)	4 (0.2)	1 (0.4)	5 (0.2)
Stomach	0 (0.0)	4 (0.2)	1 (0.4)	5 (0.2)
Kidney	2 (0.4)	1 (0.1)	0 (0.0)	3 (0.1)
Pancreas	2 (0.4)	0 (0.0)	0 (0.0)	2 (0.1)
Small bowel (duodenal)	1 (0.2)	0 (0.0)	0 (0.0)	1 (0.0)
Small bowel (ileum)	0 (0.0)	1 (0.1)	0 (0.0)	1 (0.0)
Other sites				
Lung	4 (0.7)	6 (0.3)	2 (0.7)	12 (0.5)
Melanoma	1 (0.2)	7 (0.4)	0 (0.0)	8 (0.3)
Skin	0 (0.0)	6 (0.3)	0 (0.0)	6 (0.2)
Head and neck	2 (0.4)	2 (0.1)	0 (0.0)	4 (0.2)
Brain	0 (0.0)	3 (0.2)	0 (0.0)	3 (0.1)
Lymphoma	0 (0.0)	0 (0.0)	1 (0.4)	1 (0.0)
Leukaemia	1 (0.0)	0 (0.0)	0 (0.0)	1 (0.0)
Oesophagus	1 (0.2)	0 (0.0)	0 (0.0)	1 (0.0)
Lip	1 (0.2)	0 (0.0)	0 (0.0)	1 (0.0)

*Legend*: *3D-CRT* three-dimensional conformal radiotherapy; *IMRT* Intensity modulated radiotherapy, *VMAT* volumetric modulated radiotherapy

### Impact of treatment characteristics on second tumors incidence

For the entire cohort, the calculated 10-year second tumor-free survival (STFS) in patients treated with 3D-CRT and IMRT/VMAT was 85.8 and 84.5%, respectively (*p: .627*).

**Fig. 1:**
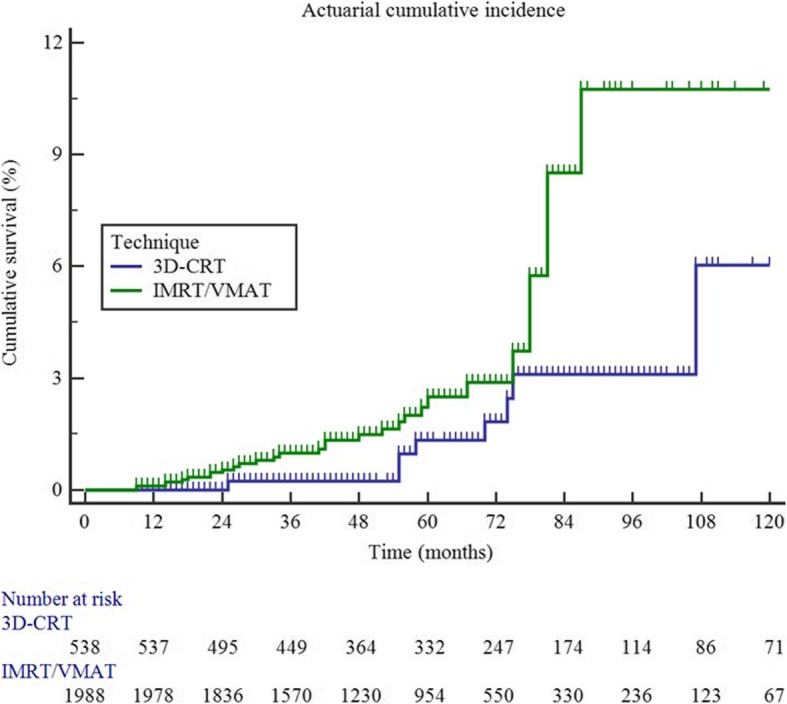
actuarial cumulative risk of pelvic second primary tumors after radiotherapy (3D- conformal therapy vs modulated techniques; *p: .033*)

At univariate analysis, 10-year STFS in patients treated with or without PNI was 84.9 and 88.1%, respectively (*p: .770*). Ten-year STFS in patients receiving or not ADT was 83.8 and 92.8%, respectively (*p: .999*). A significantly higher 10-year cumulative incidence of second tumors in the pelvis was registered in patients treated with IMRT/VMAT compared to 3D-CRT (10.7% vs 6.0%; *p: .033*). Moreover, PNI showed a trend (*p: 0.1*) for increased 10-year incidence of second tumors in both pelvis (9.4% vs 5.6%, *p: .092*) and pelvis-abdomen (10.9% vs 7.4%, *p: .064*) (Table 2**,** Fig. 1).

**Table 2:** Univariate analysis (10-year Second Tumor-Free Survival)

Variables		No. of patients (%)	all sites		pelvis		pelvis/abdomen	
			STFS	*p*	STFS	*p*	STFS	*p*
Radiotherapy technique	3D-CRT	538 (21.3)	85.8	.627	94.0	.033	92.2	.125
	IMRT/VMAT	1988 (78.7)	84.5		89.3		87.5	
Prophylactic nodal irradiation	no	1232 (48.8)	88.1	.770	94.4	.092	92.6	.064
	yes	1294 (51.2)	84.9		90.6		89.1	
Androgen deprivation therapy	no	837 (33.1)	92.8	.999	93.1	.546	92.0	.345
	yes	1689 (66.9)	83.8		91.9		89.9	
Age, years	≤ 66	688 (27.2)	85.3	.352	90.5	.981	89.6	.374
	> 66	1838 (72.8)	86.0		93.5		91.4	
Combination of radiotherapy technique and irradiated volumes	3D-CRT without PNI	326 (12.9)	91.0	.887	96.6	.044	95.3	.140
	IMRT/VMAT without PNI	906 (35.9)	78.9		89.9		87.8	
	3D-CRT with PNI	212 (8.4)	85.6		93.7		91.1	
	IMRT/VMAT with PNI	1082 (42.8)	86.6		87.6		86.3	

*Legend*: *3D-CRT* three-dimensional conformal radiotherapy, *IMRT* Intensity modulated radiotherapy, *PNI* prophylactic nodal irradiation, *STFS* second tumor free survival, *VMAT* volumetric modulated radiotherapy

**Fig. 2:**
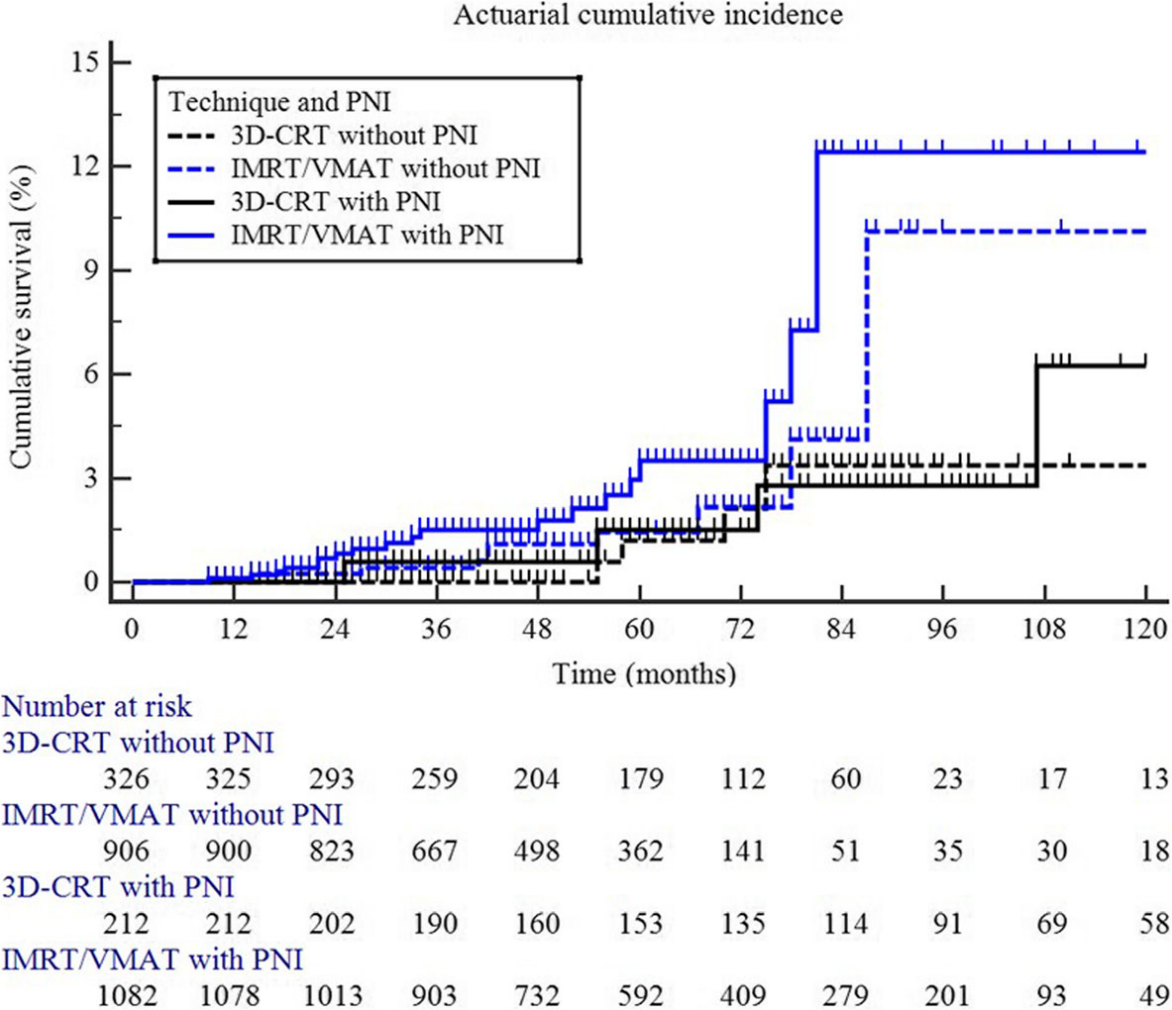
actuarial cumulative risk of pelvic second primary tumors after radiotherapy (3D-conformal radiotherapy without prophylactic nodal irradiation versus 3D-conformal radiotherapy with prophylactic nodal irradiation versus modulated radiotherapy techniques without prophylactic nodal irradiation versus modulated radiotherapy techniques with prophylactic nodal irradiation; *p: .044***)**

Stratifying patients in 4 groups according to used RT technique and irradiated volumes, a statistically significant difference was recorded in terms of STFS in the pelvis (*p: .044*). The 10-year STFS were as follows: 3D-CRT without PNI: 96.6%; 3D-CRT with PNI: 93.7%; IMRT/VMAT without PNI: 89.9%; and IMRT/VMAT with PNI: 87.6% **(**Table 2**,** Fig. 2).

On multivariate analysis **(**Table 3**)**, the lower incidence of second pelvic cancers in patients treated with 3D-CRT was confirmed (hazard ratio [HR]: 2.42, 95%CI: 1.07–5.47, *p: .034*). Furthermore, the incidence of second pelvis-abdomen cancers were found to have a trend in case of PNI delivery (HR: 1.63, 95%CI: 0.95–2.79, *p: .067*). Moreover, in a separate multivariate analysis where RT techniques and irradiated volumes were combined, patients treated with IMRT/VMAT plus PNI were found to have a significantly increased risk of second pelvic cancers (HR: 3.24, 95%CI: 1.09–9.65, *p: .035*) and second pelvis-abdomen cancers (HR: 2.61, 95%CI: 1.06–6.41, *p: .037).* The Fine and Gray regression model analysis confirmed a trend of influence of IMRT/VMAT on the incidence of pelvic tumors (p: 0.058). Furthermore, considering the combination of radiotherapy technique and irradiated volumes, the same model confirmed the significant impact of IMRT/VMAT plus PNI on pelvic (p: 0.033) and pelvic or abdominal (p: 0.034) second tumors, using 3D-CRT without PNI as a reference.

**Table 3:** Multivariate analysis on second tumor free survival

*Variable*	*value*	*pelvic*		*Pelvic-abdominal*			
		*HR*	*95%CI*	*p*	*HR*	*95%CI*	*p*
Radiotherapy technique	3D-CRT	Ref					
	IMRT/VMAT	2.42	1.07–5.47	.034			
Prophylactic nodal irradiation	No				Ref		
	Yes				1.63	0.95–2.79	.067
Combination of radiotherapy technique and irradiated volumes	3D-CRT without PNI	Ref			Ref		
	IMRT/VMAT without PNI	1.70	0.53–5.51	.375	1.66	0.64–4.29	.294
	3D-CRT with PNI	1.10	0.27–4.46	.892	1.73	0.61–4.92	.303
	IMRT/VMAT with PNI	3.24	1.09–9.65	.035	2.61	1.06–6.41	.037

*Legend*: *3D-CRT* three-dimensional conformal radiotherapy, *IMRT* Intensity modulated radiotherapy, *PNI* prophylactic nodal irradiation, *VMAT* volumetric modulated radiotherapy

## Discussion

We performed an analysis on the incidence of second cancers in PCa patients treated with EBRT to evaluate the impact of RT technique, irradiated volumes, and ADT. The analysis showed a significant correlation between 10-year incidence of second tumors located in the pelvis and RT technique [3D-CRT vs IMRT (6.0% vs 10.7%, *p: .033*)], while PNI showed a trend for increased 10-year incidence of second tumors in both pelvis (9.4% vs 5.6%, p: .092) and pelvis-abdomen (10.9% vs 7.4%, p: .064).

Our study has several limitations which simply make it a hypothesis generating analysis. First, the median follow-up is relatively short (72 months). In fact, in a cohort of Hodgkin’s Lymphoma patients treated with RT, the median latency time to second tumor was 7.5 years [23]. We cannot completely exclude that with a longer follow-up, the recorded differences could lose their statistical significance. Furthermore, not having defined a minimum follow-up duration as an inclusion criterion, it is possible that some of the second malignancies diagnosed during the follow-up had arisen before RT. This aspect may have influenced the results of the analysis. Moreover, the sample size (2526 patients) can be considered relatively small. In fact, other studies in this field [3, 6, 24], two of which were registry studies [3, 24], included 9538–619,479 patients. Even this small sample size could potentially have affected the results of the analysis. Furthermore, although image guided RT could add a non-negligible risk for second tumors when daily set-up verification with high-resolution modality is performed [13], we did not consider this issue in our analysis. However, it should be noted that no extra dose was delivered for set-up verification in most patients. Furthermore, only a small minority of patients treated in the last 2 years had their treatment position and organ motion checked using a daily cone-beam CT. In addition, the evaluation of other potential factors correlated with second tumors are lacking in our analysis. For example, the first 2 primary tumors recorded in this study were bladder and lung cancers and both are smoking-related malignancies. Therefore, it would have been interesting to evaluate the impact of RT techniques also considering the smoking habits of individual patients. Unfortunately, even in this case, this data is only available in a minority of patients and therefore could not be analysed. Moreover, it should be noted that until 2009 (when all patients of the 3D-CRT group were treated), the cranial margin for pelvic nodes delineation was at the level of the anterior margin of the S1 vertebra. From 2009 (when all patients were treated with modulated techniques), this margin was moved slightly cranially, at the level of the S1-L5 interspace. Therefore, we cannot rule out that most patients undergoing modulated techniques received a slightly more extensive irradiation in the cranial direction and that this may have influenced the results of our analysis.

Finally, patients with short observation time were not excluded in order to consider a reasonable latency time between RT and onset of the second tumor. For example, in a previous study, the analysis of second solid cancers was based only on 5-year survivors and analysis of leukemia were based only on 2-year survivors [25]. However, given the uncertainty about the latency times of second tumors occurrence, we decided to use a conservative criterion and therefore to include all primitive tumors diagnosed after RT.

In the past, even if the results are somehow contradictory [26] and the incidence of second tumors could also be attributed to age and lifestyles [6], several analyses showed an increased risk for second tumors after EBRT of PCa [3–5, 24]. Probably these data should be considered with caution. In fact, in previously cited studies [3–5, 24], the incidence of second tumors was evaluated by comparing PCa patients who underwent RT with subjects receiving other treatments, mainly represented by radical prostatectomy (RP). In this regard, it should be noted that in different risk categories, RT and RP are considered as alternative therapeutic options. However, in daily clinical practice, the choice between the two treatments is often based on patient’s comorbidities. In particular, RT is preferred to RP in case of contraindications to surgery. These contraindications (COPD, cardiovascular diseases, metabolic syndrome) are more frequent in smoking patients and these subjects are obviously more prone to smoke-related malignancies such as bladder or lung tumors.

Some studies evaluated also the impact of RT technique on the incidence of second tumors. In particular, three meta-analyses uniformly recorded a higher incidence of second rectal tumors after EBRT but not after brachytherapy [4, 5, 26]. In another study no differences were observed in terms of overall incidence of second tumors between 2D-conventional and 3D-CRT but only an advantage in patients undergoing 3D-CRT in terms of second rectal tumors. In the same analysis, no significant differences were observed based on beams photons energy (> 10 MV versus ≤10 MV) but a reduction in colon and leukaemia tumors in patients undergoing brachytherapy compared to those treated with external beams [25].

However, to the best of our knowledge, our study is the first analysis comparing 3D-CRT vs IMRT/VMAT techniques and evaluating also the impact of PNI and ADT. Furthermore, we considered the incidence of second tumors in different body regions (pelvis, pelvis or abdomen, and all together). The results of our analysis based on clinical data are in agreement with several dosimetric and planning studies predicting a higher incidence of bladder and/or rectal second cancers in patients treated with modulated techniques [15–18].

More generally, our study showed a 14.4% 10-year incidence of second tumors. Considering the favourable prognosis related to PCa (10-year OS: 87.3% in our series), this result should stimulate attention during the follow-up of patients not only to eventual PCa relapse but also to the risk of second tumors. In particular, haematuria or rectal bleeding should not be automatically considered as late RT induced toxicity but should also lead to further investigations on the possibility of bladder or rectal cancer, respectively.

Given the increased risk of radiation induced second tumors in PCa patients receiving RT, this possibility should be discussed with patients before treatment [3]. Based on our analysis, not showing a significant increase in the overall incidence of second cancers, further explanations about the potential additional risk from modulated RT techniques seem not required.

However, further analysis with prolonged follow-up, possibly on larger patients’ population and considering other risk factors such as smoking habits, should be performed to confirm our findings.

## Conclusions

The results of our study suggest a correlation between the use of modulated techniques and the incidence of second pelvic tumors in RT of PCa. Furthermore, the combination of modulated techniques and PNI seems to increase the incidence of second abdominal-pelvic tumors. These results appear relevant given that: i) they justify the planning of further studies on this topic; ii) if confirmed by these future analyses, they would represent the first “clinical” confirmation of the hypothetical impact of modulated techniques on the incidence of second tumors; iii) these results suggest particular attention, in the follow up of patients treated with modulated techniques, to the early diagnosis of second pelvic tumors and, in case of PNI, also of second abdominal tumors.

## Data Availability

The datasets used and/or analysed during the current study are available from the corresponding author on reasonable request.

## Abbreviations

3D-CRT: 3-dimensional conformal RT
ADT: Androgen deprivation therapy
CT: Computed tomography
CTV: Clinical Target Volumes
EBRT: External beam RT
IMRT: Intensity-modulated RT
OAR: Organs at risk
PCa: Prostate cancer
PNI: Prophylactic nodal irradiation
RT: Radiotherapy
STFS: Second tumor free survival
VMAT: Volumetric modulated arc therapy
